# Cystic echinococcosis in patients with leukemia: Clinical challenges and review of reported cases

**DOI:** 10.1016/j.ijregi.2026.100840

**Published:** 2026-01-08

**Authors:** Karel Santamaria-Leandro, Bruno Guerrero-Arismendiz, César Castro-Prado, Giancarlo Pérez-Lazo, Wilmer Silva-Caso

**Affiliations:** 1 Dirección de Redes Integradas de Salud, Lima Este, Peru; 2 Clinical Hematology Department, Guillermo Almenara Irigoyen National Hospital-EsSalud, Lima, Peru; 3 Division of Infectious Diseases, Guillermo Almenara Irigoyen National Hospital-EsSalud, Lima, Peru; 4 Biomedicine Laboratory, Research Center of the Faculty of Health Sciences, Universidad Peruana de Ciencias Aplicadas, Lima, Peru

**Keywords:** Cystic echinococcosis, Leukemia, Immunocompromised, Peru

## Abstract

**Objectives:**

Cystic echinococcosis (CE) rarely coexists with hematologic malignancies. We report two Peruvian men with leukemia and CE.

**Case report:**

One patient had chronic lymphocytic leukemia and a hepatic CE2 cyst managed with albendazole and surgery before chemoimmunotherapy, and another patient had B-cell acute lymphoblastic leukemia and prior hepatic and pulmonary CE treated with concurrent albendazole and induction chemotherapy. Both patients lived in endemic highland areas and had childhood exposure to dogs.

**Discussion:**

A literature review identified only a few cases, mostly acute myeloid leukemia, and none from Latin America.

**Conclusion:**

These cases highlight therapeutic dilemmas in balancing infection control and malignancy treatment, underscoring the need for tailored management strategies in endemic regions.

## Highlights

• Two rare cases of cystic echinococcosis in patients with leukemia.
• Reports are scarce, and cases from Latin America appear to be underrepresented.
• Albendazole was administered with chemotherapy without clinically significant hepatic toxicity.
• Antiparasitic and oncologic treatment depended on leukemia type and the patient’s stability.
• Individualized approaches to parasitic infections in immunocompromised hosts.

## Introduction

Cystic echinococcosis (CE), a zoonotic infection caused by *Echinococcus granulosus*, remains endemic in the Peruvian Andes and other livestock-raising regions [[1], [2], [3]]. CE in immunocompromised hosts is uncommon, and its coexistence with hematologic malignancies is exceedingly rare. The interaction between parasite biology and host immune suppression remains poorly characterized, and the optimal sequence of antiparasitic and oncologic treatments is uncertain [[4], [5], [6], [7], [8], [9]]. We present two cases of CE and leukemia with contrasting hematologic profiles and outcomes, along with a concise review of the literature.

## Case presentation

### Case 1

**Figure 1:**
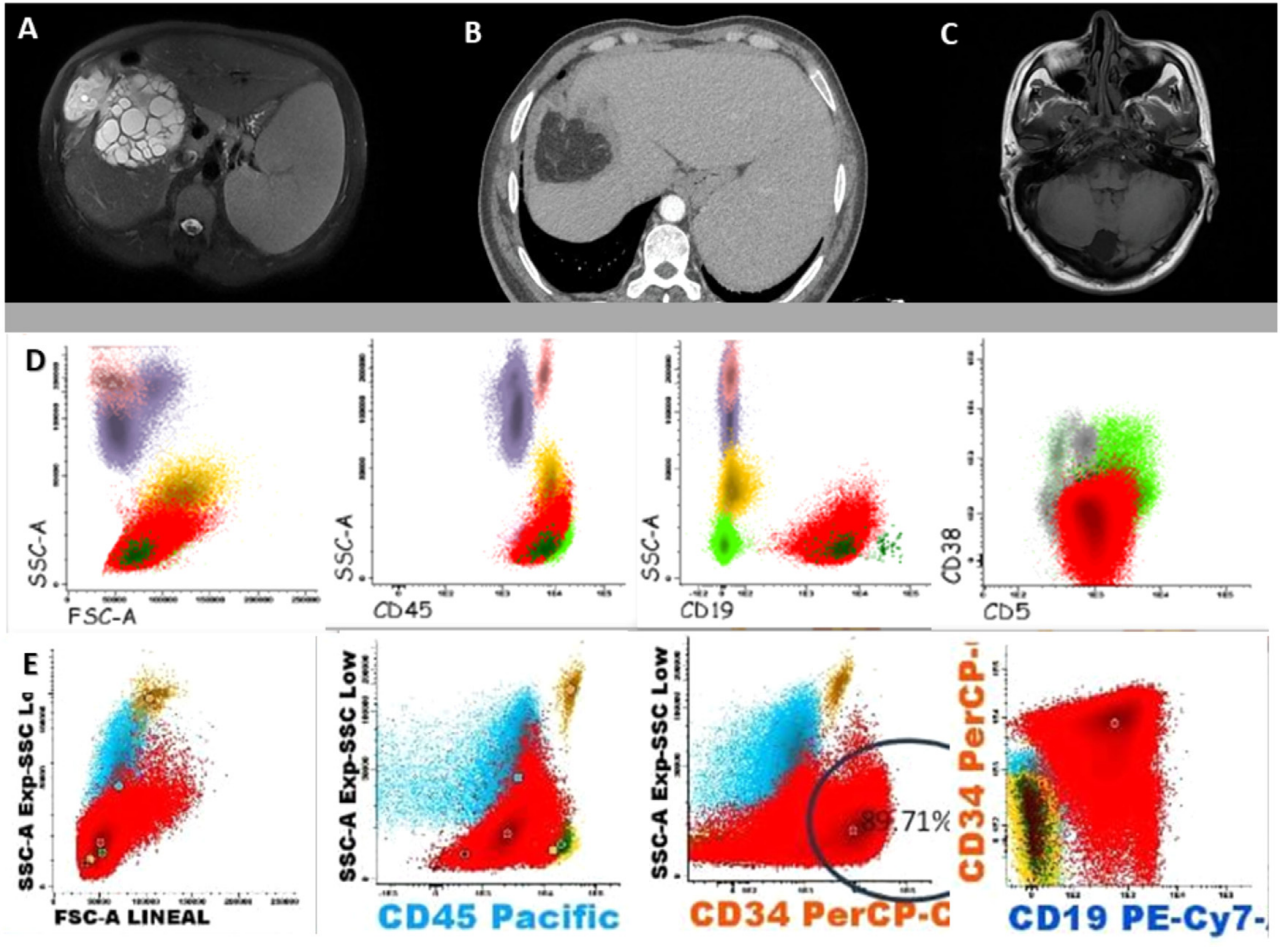
Imaging and immunophenotypic findings. (a) Abdominal magnetic resonance (August 24, 2022) imaging showing a large heterogeneous hepatic mass (129 × 163 × 143 mm) in the right lobe, with multiple daughter cysts and a peripheral hypointense capsule, findings consistent with a hepatic hydatid cyst (Case 1). (b) Abdominal CT (June 5, 2023) showing hepatomegaly with focal defect and absence of segment VIII, with an adjacent cystic image of ∼26 mm, consistent with postoperative changes. Splenomegaly is also observed (Case 1). (c) Axial T1-weighted spin echo brain magnetic resonance imaging without contrast showing a well-defined cystic lesion in the posterior fossa, consistent with an infratentorial arachnoid cyst, with mild displacement of the left cerebellar hemisphere (Case 2). (d) Flow cytometry of peripheral blood showing an aberrant mature B-cell population expressing CD19 and CD5, consistent with chronic lymphocytic leukemia (Case 1). (e) Flow cytometry of peripheral blood showing a blast population with low CD45 expression and positivity for CD34 and CD19, consistent with B-cell acute lymphoblastic leukemia (Case 2).

A 55-year-old man from Cerro de Pasco, in the central highlands of Peru, with a medical history of von Willebrand disease and rheumatoid arthritis, presented with a 6-month history of fatigue, asthenia, and unintentional weight loss. Physical examination revealed cervical lymphadenopathy, gingival bleeding, and hepatosplenomegaly. Laboratory tests showed marked leukocytosis (53.6 × 10^3^/µL) with absolute lymphocytosis (49.29 × 10^3^/µL), anemia (hemoglobin: 9.5 g/dL; hematocrit: 30.4%), and severe thrombocytopenia (17.6 × 10^3^/µL). Flow cytometry confirmed the diagnosis of chronic lymphocytic leukemia (CLL) (Figure 1). Abdominal magnetic resonance imaging revealed a large right-lobe hepatic cyst with multiple daughter vesicles, classified as Gharbi type III/World Health Organization (WHO) CE2, with positive *E. granulosus* serology (immunoglobulin G enzyme-linked immunosorbent assay: 16.4 µg/mL; reference <9 µg/mL) (Figure 1). The patient received albendazole (800 mg/day), followed by surgical unroofing of the cyst. Six months later, chemotherapy with fludarabine, cyclophosphamide, and rituximab was initiated, and complete remission of CLL was achieved after 3 cycles. The subsequent course was favorable, with no recurrence of the disease. One year later, the patient died of intestinal subocclusion, with no evidence of echinococcosis recurrence and stable hematological parameters at follow-up.

CD, clusters of differentiation.

### Case 2

A 45-year-old man from Huancavelica, in the central highlands of Peru, had a history of pulmonary CE diagnosed 3 years earlier, managed by right thoracotomy with cyst evacuation, closure of bronchial fistulas, capitonnage, and chest drainage. He received albendazole for 2 months preoperatively and 9 months postoperatively, with favorable outcomes. Seven months before the current presentation, he underwent hepatic CE surgery involving segment VIII, consisting of adhesiolysis, aspiration, and inactivation of the cyst with a scolicidal solution, membrane removal, and drainage, followed by 5 months of albendazole. The therapy was discontinued 3 weeks prior to admission. He presented with a 2-month history of holocranial headache, malaise, nausea, and mild abdominal discomfort. On admission, the examination revealed pallor and neck stiffness. Laboratory testing revealed hemoglobin 15.1 g/dL, hematocrit 44.6%, platelets 50 × 10^3^/µL, and leukocytosis (121 × 10^3^/µL) with neutropenia (1.08 × 10^3^/µL) and lymphocytosis (8.4 × 10^3^/µL). Flow cytometry confirmed B-cell acute lymphoblastic leukemia (B-ALL) with both pro-B and common B-cell immunophenotypes (Figure 1). Brain magnetic resonance imaging revealed a 3 mm calcification in the left frontal subcortical white matter and a posterior fossa arachnoid cyst (Figure 1). Cerebrospinal fluid (CSF) cultures for bacteria, fungi, and mycobacteria as well as CSF flow cytometry, were negative, but Western Blot for *E. granulosus* was positive; however, this finding alone was insufficient to confirm central nervous system involvement, particularly in a patient with a history of multiorgan CE. Given the aggressive course of leukemia, albendazole 800 mg/day was initiated concurrently with induction chemotherapy (vincristine, daunorubicin, prednisone, and L-asparaginase). The regimen was well tolerated without treatment-related adverse events, but no hematologic remission was achieved. Palliative methotrexate and prednisone were administered; however, the patient died of a hemorrhagic stroke. In the absence of pathognomonic neuroimaging features and without histopathological confirmation, the diagnosis was considered probable neurohydatidosis, based on the patient’s epidemiological background, prior hepatic and pulmonary CE, compatible neurological symptoms, and positive CSF serology.

Both patients had contact with unvaccinated dogs during childhood.

## Discussion

The coexistence of CE and leukemia is exceptionally rare, with most published cases involving acute myeloid leukemia (AML) [[4], [5], [6],^8^]. Reports of CE associated with CLL or B-ALL are scarce, and cases from Latin America appear to be underrepresented in the published literature. The available literature describes variable approaches to therapy, often prioritizing chemotherapy over antiparasitic management because of the risk of cyst rupture or secondary infection during aplasia [4]. However, in indolent malignancies such as CLL, early control of the parasitic focus may prevent complications during immunosuppression.

Ghasemirad et al. [9] reviewed 210 immunocompromised cases of echinococcosis, identifying human immunodeficiency virus (HIV) infection as the most frequent underlying condition, while only 1.3% involved hematologic neoplasms. Alveolar echinococcosis predominates in immunosuppressed European patients, whereas cystic forms remain endemic in low- and middle-income regions. Peru reports 1 of the highest burdens of CE in South America, accounting for over 20,000 human cases between 2009 and 2014, mostly from the Andean highlands of Arequipa, Cusco, Huancavelica, Junín, Pasco, and Puno. Transmission remains driven by dog–livestock cycles, informal slaughtering, and limited rural sanitation, resulting in substantial economic losses and persistent endemicity despite available control tools [10,11].

Most published reports of concomitant CE and leukemia involve AML, a biologically aggressive malignancy that typically requires urgent initiation of induction chemotherapy. In this context, antiparasitic management has frequently been deferred or omitted. For example, Ali et al. [4] reported a 19-year-old male from Turkey with AML and a hepatic CE1 cyst, in whom chemotherapy was prioritized; no antiparasitic treatment was administered, and the outcome was fatal due to the underlying disease. Similarly, Singh et al. [8] described a 36-year-old male from India with concomitant AML and hepatic CE1 cysts, in whom chemotherapy was initiated first, followed by percutaneous drainage of the cysts after achieving complete remission. In another case, Kim et al. [5] reported a 65-year-old female from Armenia with AML and multivesicular hepatic cysts, in whom surgical management with left hepatectomy was required after hematopoietic stem cell transplantation, as the cysts remained stable in size. Collectively, these reports illustrate that in aggressive leukemias such as AML, the biological behavior and immediate mortality risk of the malignancy often dictate therapeutic sequencing, with chemotherapy taking precedence over antiparasitic control.

Central nervous system involvement in CE is rare, accounting for approximately 2-3% of reported cases, and its diagnosis relies primarily on characteristic neuroimaging findings and, when feasible, surgical or histopathological confirmation [12]. Cerebral CE typically presents as solitary, well-defined, supratentorial cystic lesions associated with symptoms of increased intracranial pressure. In contrast, small calcifications, arachnoid cysts, or nonspecific magnetic resonance imaging findings are not considered diagnostic, and serological assays may only support prior exposure, particularly in patients with a history of multiorgan echinococcosis [12]. Accordingly, in our second case, the absence of pathognomonic imaging features and lack of confirmatory procedures warranted a cautious interpretation, and the diagnosis was classified as probable neurohydatidosis rather than confirmed disease.

According to WHO guidelines, uncomplicated hepatic CE1-CE3a cysts are managed medically with albendazole (400 mg twice daily for 3-6 months), whereas larger cysts may require percutaneous drainage or puncture, aspiration, injection, and reaspiration (PAIR) in combination with antiparasitic therapy [13]. In immunocompromised hosts, surgery combined with albendazole is common because of disease severity [9]. Although concerns regarding potential hepatotoxicity often limit the use of albendazole during chemotherapy [14], both of our patients received combined treatment without evidence of clinically significant hepatic toxicity.

In case 1, antiparasitic therapy and surgical control were prioritized over chemoimmunotherapy because of the indolent course of CLL. Once progression criteria appeared, standard fludarabine, cyclophosphamide, and rituximab therapy was initiated, achieving remission [15]. Case 2 exemplifies the difficulty in treating aggressive ALL with concurrent CE and probable neurohydatidosis. Albendazole was administered alongside induction chemotherapy, yet the outcome was poor, reflecting treatment-related toxicity typical in Latin American settings [16]. These cases underscore the need for individualized protocols balancing parasitic control, chemotherapy intensity, and supportive care, together with long-term follow-up, given the risk of CE recurrence.

## Conclusion

In cases of concomitant CE and leukemia, therapeutic sequencing should be tailored to the biology and urgency of the malignancy. Though hydatid management may be prioritized in indolent neoplasms (e.g., CLL) and leukemia-directed therapy in aggressive forms (e.g., ALL), these conclusions are based on limited evidence and should be confirmed by future studies.

## Declaration of competing interest

The authors have no competing interests to declare.
